# Amphiphilic dendritic peptides: Synthesis and behavior as an organogelator and liquid crystal

**DOI:** 10.3762/bjoc.7.26

**Published:** 2011-02-11

**Authors:** Baoxiang Gao, Hongxia Li, Defang Xia, Sufang Sun, Xinwu Ba

**Affiliations:** 1 Key Laboratory of Medicinal Chemistry and Molecular Diagnosis, Ministry of Education, College of Chemistry and Environment Science, Hebei University, Baoding 071002, P.R. China

**Keywords:** amphiphilic, dendritic peptides, liquid crystal, organogels

## Abstract

New amphiphilic dendritic peptides on dendritic polyaspartic acid were designed and synthesized. The organogel and liquid crystal properties of these amphiphilic dendritic peptides were fully studied by field-emission SEM, temperature dependent FT-IR, differential scanning calorimetry, polarization optical microscopy and X-ray diffraction experiments. Amphiphilic dendritic peptides **G3** show good organogel properties with a minimum gelation concentration as low as 1 wt %. Furthermore, amphiphilic dendritic peptides **G3** can form a hexagonal columnar liquid crystal assembly over a wide temperature range.

## Introduction

Peptide self-assembly has drawn a significant attention due to potential applications, especially in the fields of biomedicine and bionanotechnology [1–3]. Programmed self-assembly of peptides into highly ordered nanostructures creates biomaterials that display a wide range of physical properties often exceeding those of synthetic polymers [4–5]. Peptide-amphiphiles (PAs) represent an attractive class of bioactive molecules as they self-assemble into a variety of nanostructures, many of which have promising biological activity due to the exposed peptide regions on their outer surface [6–7]. The self-assembly of amphiphilic oligopeptide systems is thus emerging as a particularly powerful strategy to direct the self-assembly of relatively simple peptide building blocks toward sophisticated nanostructures [8]. Furthermore, the natural amino acid based dendrons or dendrimers are of great significance because of their similarity to proteins in composition and topology [9–11], as well as their architectural difference from currently prevalent linear model peptides. However, little attention has been paid to the self-assembly of natural amino acid based dendrimers, and especially to their gelation and liquid crystal properties [12–13]. Recently, a type of amphiphilic dendritic dipeptide was described by Percec et al. as self-assembling in helical pores [14]. In addition, Takashi Kato and co-workers have recently reported that dendritic oligopeptides can act as useful building blocks for chiral supramolecular liquid crystals [15–16].

Herein, we present the synthesis of amphiphilic dendritic peptides (ADPs) composed of an aspartic acid core and an aliphatic periphery, and their self-assembly which leads not only to organogels but also to liquid crystals.

## Results and Discussion

### Synthesis and characterization

**Scheme 1:**
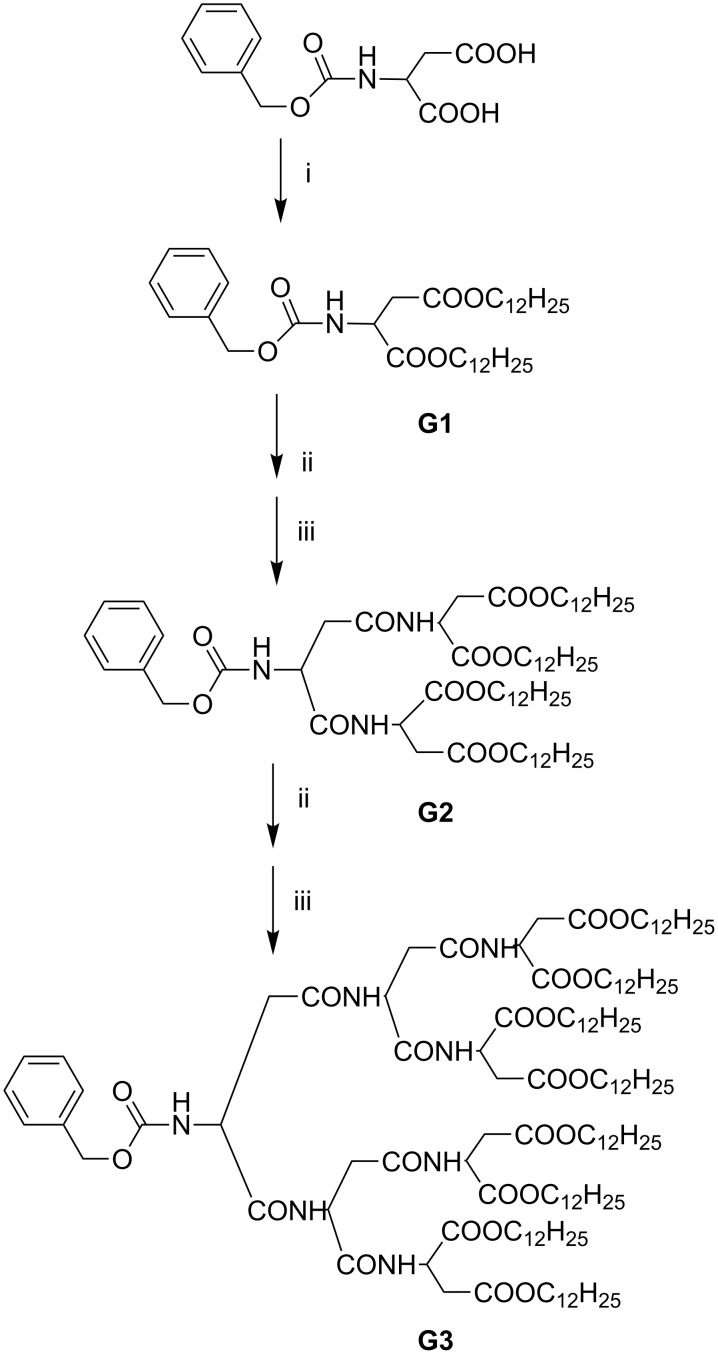
Synthetic sequence of amphiphilic dendritic peptides: (i) 1-dodecanol, EDCI, DMAP, dichoromethane, 25 °C. (ii) H_2,_ Pd/C, ethanol, 25 °C. (iii) *N*-carbobenzyloxy-L-aspartic acid, EDCI, HOBt, dichoromethane, 25 °C.

The amphiphilic dendritic peptides were synthesized convergently as depicted in Scheme 1 via standard EDCI coupling of *N*-carbobenzyloxy-L-aspartic acid and L-aspartic acid dodecyl ester (readily prepared by the esterification of aspartic acid with 1-dodecanol). The second (**G2**) and third generations (**G3**) were synthesized convergently in 75 and 60% yields, respectively, by repeating a two-reaction cycle, i.e., by removal of the carbobenzyloxy group of the lower-generation dendron by hydrogenation, and then coupling the resulting *N*-deprotected intermediate to the *C*-deprotected **G1**, prepared by hydrogenolysis of the benzylated peptide. ^1^H NMR, MALDI-TOF mass spectrometry, and elemental analyses were used to verify the structure and purity of the amphiphilic dendritic peptides.

### Investigation of gelation

All amphiphilic dendritic peptides were subjected to gelation tests in a variety of organic solvents. Each compound was combined with solvent in a screw-capped sample tube and heated until the solid completely dissolved. The solution was then cooled to room temperature, and gelation monitored by inverting the sample tube to see whether the solution flows or not. The observed gelation behavior of each solution is summarized in Table 1. Compound **G1** showed good solubility in a range from non-polar (*n*-hexane) to polar solvents (ethanol), and showed no gelation in any solvent tested. Compound **G2** can gel only in the nonpolar *n*-hexane. Compound **G3** gelled in *n*-hexane, toluene, 1,4-dioxane, and ethanol but was soluble in dichloromethane.

**Table 1:** Gelation of organic solvents by the amphiphilic dendritic oligopeptides^a^.

	*n*-hexane	toluene	dichloromethane	1,4-dioxane	ethanol

**G1**	S	S	S	S	S
**G2**	G (cgc = 3%)	S	S	S	S
**G3**	G (cgc = 1%)	G (cgc = 5%)	S	G (cgc = 6%)	G (cgc = 2%)

^a^cgc: gelation concentration (w/w %). G: gel. S: solution.

**Figure 1:**
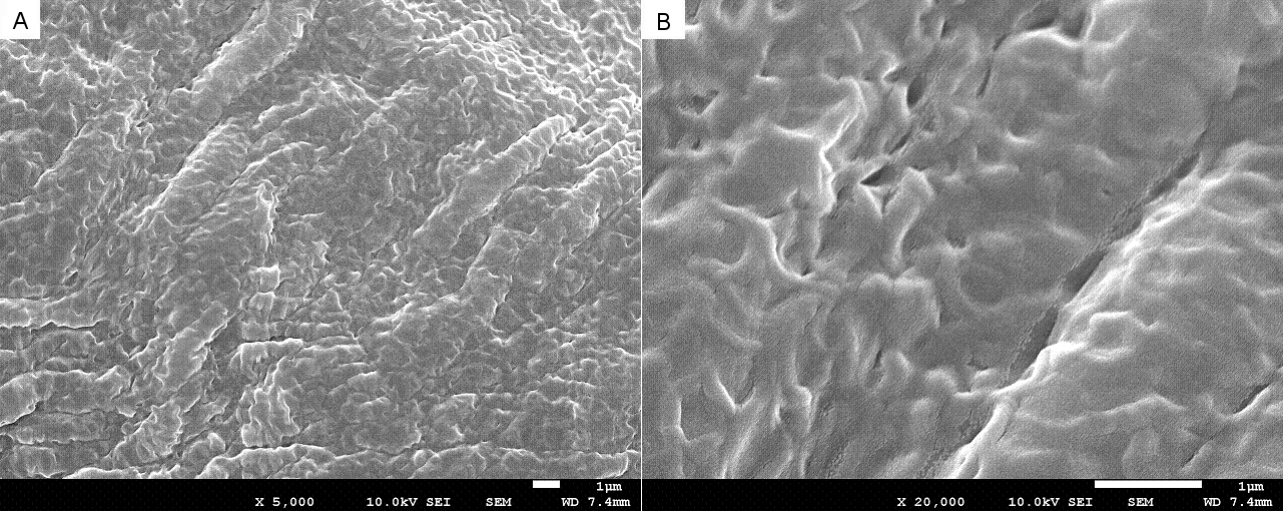
(A) SEM images of the xerogels of **G3** from *n*-hexane (B) magnification of the xerogels structure.

The microscopic structure of the gel was investigated by field-emission SEM (FE-SEM). Figure 1 shows representative FE-SEM images of the xerogel of **G3** formed in *n*-hexane. These images clearly show that organogels assembled from **G3** formed thin fibres that underwent further aggregation to form fibre bundles. These fibre bundles constitute a highly developed entangled network.

**Figure 2:**
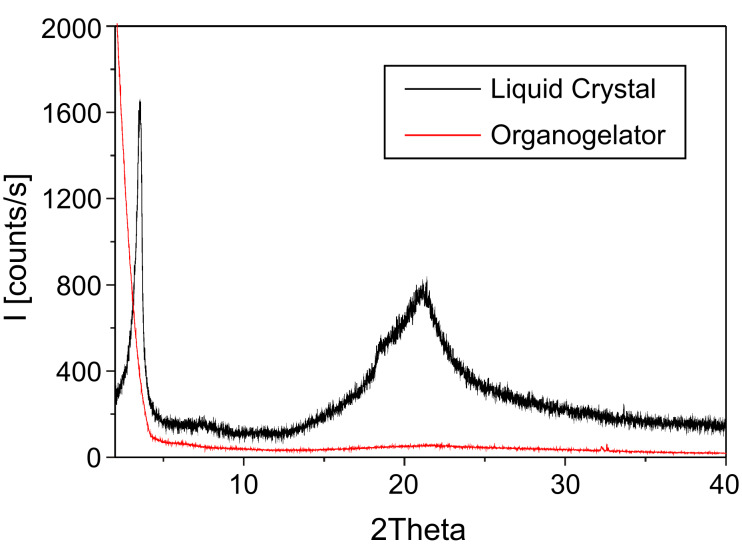
X-ray diffraction patterns of **G3** as an organogelator and liquid crystal.

**Figure 3:**
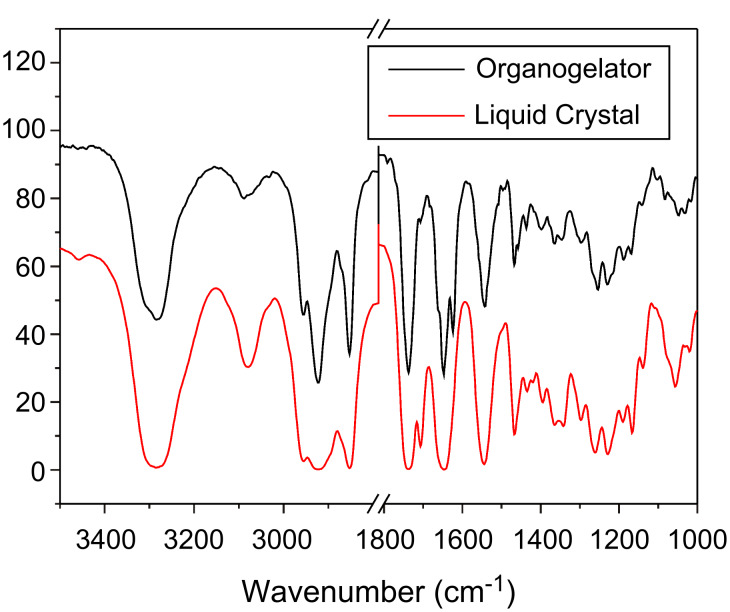
FT-IR of **G3** as an organogelator and liquid crystal.

Figure 2 shows the XRD scan of organogelator **G3** and liquid crystal **G3**. Being different from the liquid crystal, the XRD of organogelator shows no reflection peaks in the small-angle region which indicates that molecular arrangement in the organogelator is disordered. Figure 3 shows the FT-IR of **G3** as an organogelator and as a liquid crystal. The absorption at 3081 cm^−1^ is ascribed to the N–H stretching frequency of the hydrogen bonds. The absorption intensity at 3081 cm^−1^ decreases in the organogelator which indicates the loss of hydrogen bonds.

Hydrogen bonds as physical cross-linking points in these compounds are an essential factor for gelling. With more physical cross-linking points, compound **G3** gels more easily. Compound **G2** can gel only in *n*-hexane. Compared with the other solvents, *n*-hexane is a poor solvent, in which hydrogen bonds form more easily. It is therefore reasonable that the minimum gelation concentration of **G3** (1 wt %) was lower than **G2** (3 wt %) in the same (*n*-hexane) solvent.

### Investigation of the liquid crystalline behavior

**Figure 4:**
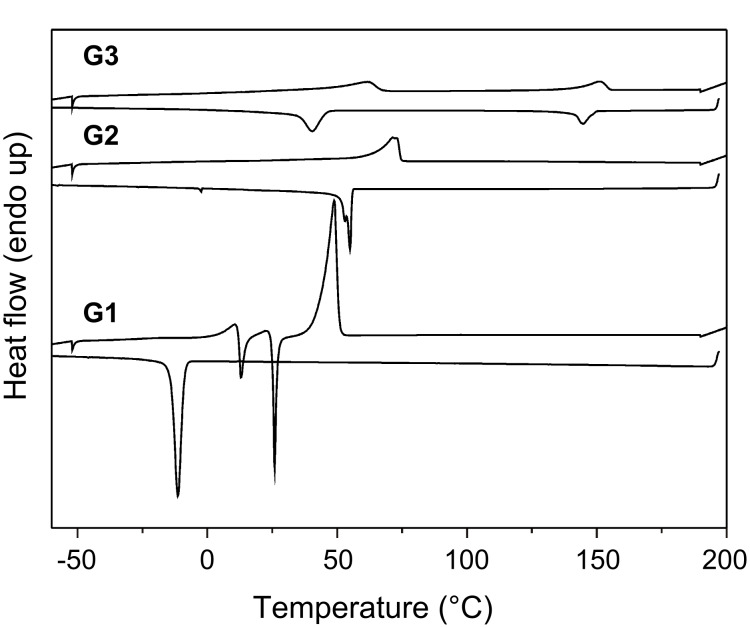
Differential scanning thermograms of ADPs registered during the second heating–cooling cycle with scanning rate 10 K min^−1^.

Thermotropic behavior of ADPs was investigated by a combination of differential scanning calorimetry (DSC), polarization optical microscopy (POM) and X-ray diffraction (XRD) experiments. Figure 4 shows DSC curves of amphiphilic dendritic peptides. In the DSC scan from −60 °C to 200 °C, **G1** showed three phase transitions at 15 °C, 25 °C and 40 °C, which are attributed to phase transitions of recrystallization and crystal to isotropic melt. **G2** only showed a phase transitions at 71 °C, which is attributed to the phase transition of crystal to isotropic melt. DSC analysis of **G3** showed phase behavior, where a LC mesophase, on a second cooling, appeared at 40 °C and then disappeared at 145°C to form an isotropic melt (Figure 4). The high temperature for the isotropic melt of **G3** is the result of hydrogen bond interactions of the amide groups which reinforce the columnar organization.

**Figure 5:**
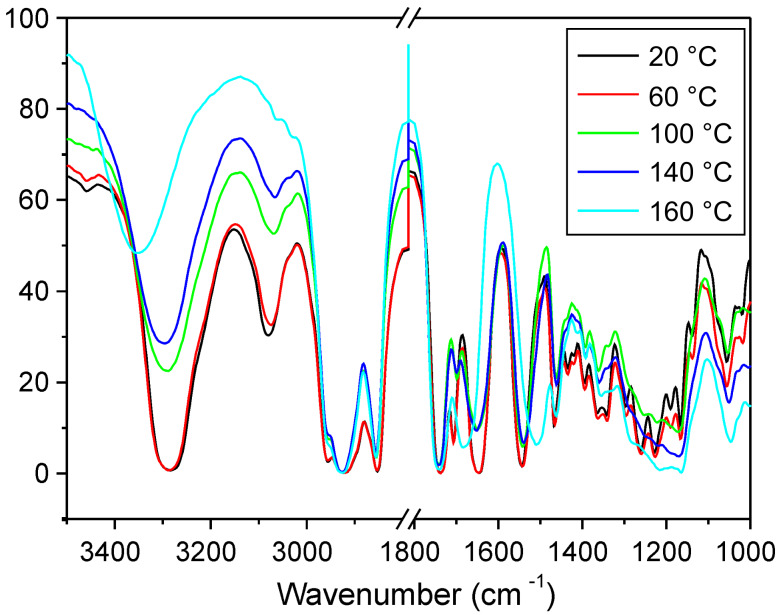
Temperature dependent FT-IR of **G3**.

The presence of hydrogen bonds in the liquid-crystalline phases of **G3** is demonstrated by temperature dependent FT-IR studies (Figure 5). Cooling from 160 °C (isotropic state) to 20 °C (liquid crystalline state), the absorption intensities of the free amide groups at 1685 cm^−1^ decrease. The absorption intensities of the hydrogen bonding amide groups 1648 cm^−1^ increase. Furthermore, the N–H stretching frequency of **G3** shifts from 3354 cm^−1^ (160 °C) to 3288 cm^−1^ (20 °C), and the absorption intensities at 3084 cm^−1^ (the N–H streching frequency of the hydrogen bonds) also increase which indicates that hydrogen bonds form in liquid crystalline. With the high selectivity and directionality of hydrogen bonds, the formation of the hydrogen bonds should contribute to the columnar liquid crystalline properties.

**Figure 6:**
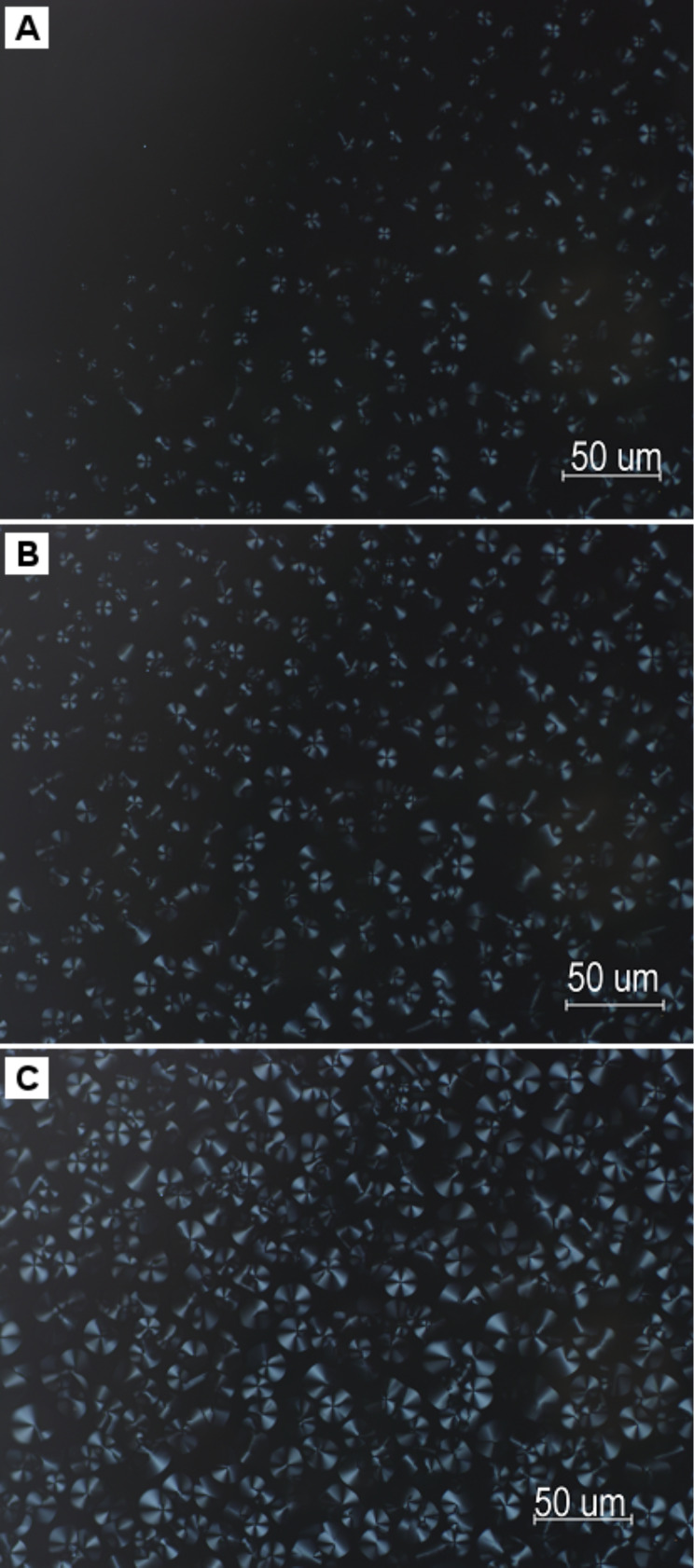
. Polarized optical micrographs of **G3** at 145 °C (A), 140 °C (B), and 50 °C (C).

**Figure 7:**
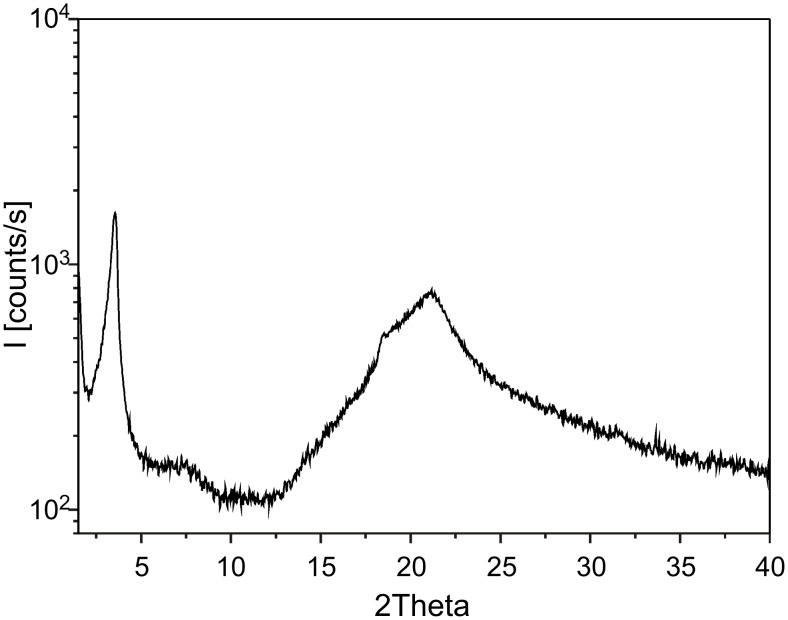
X-ray diffraction patterns of **G3** at 50 °C.

Cooling from 160 °C to 145 °C, **G3** begins to form the conic fan-shaped textures (Figure 6A), which are characteristic of hexagonal columnar liquid crystals. The conic fan-shaped textures of **G3** grow (Figure 6B), and no longer change at 50 °C (Figure 6C). Figure 7 shows the XRD scan for the birefringent phase of **G3** at 50 °C. The birefringent phases of **G3** were further confirmed as a hexagonal columnar liquid crystal phase through assignment of the reflections. In the wide angle region, the diffused halo at around 4.5 Å is due to the disorder of the terminal alkyl chains. In the small-angle region, the XRD profile of **G3** shows two reflection peaks corresponding to d spacing of 24.8 and 12.5 corresponding to (100) and (200) reflections. The XRD results show the compound **G3** self-assembles into columnar structures.

## Conclusion

In summary, we designed and synthesized amphiphilic peptides on dendritic polyaspartic acid. These new amphiphilic dendritic peptides are capable of gelling a variety of organic solvents to form stable organogels via cooperative non-covalent forces, with a minimum gelation concentration as low as 1 wt %. It is interesting that amphiphilic dendritic peptides **G3** can form a hexagonal columnar liquid crystals over a wide temperature range.

## Experimental

L-Aspartic acid and *N*-carbobenzyloxy-L-aspartic acid were purchased from Yangzhou Baosheng Bio-Chemical Co. Ltd. *N*-(3-Dimethylaminopropyl)-*N*'-ethylcarbodiimide hydrochloride (EDCI), 4-dimethylaminopyridine and 1-hydroxy benzotriazole were purchased from Shanghai Medpep Co. Ltd. 1-Dodecanol and solvents were purchased from Sinopharm Chemical Reagent Co. Ltd, and used without any further purification. Solvents used for precipitation and column chromatography were distilled under normal atmospheric pressure. ^1^H NMR spectra were recorded at 20 °C on a 300 MHz NMR spectrometer (Bruker). Chemical shifts are reported in ppm at room temperature in CDCl_3_ with tetramethylsilane as internal standard unless indicated otherwise. Mass spectra were carried out using MALDI-TOF/TOF matrix assisted laser desorption ionization mass spectrometry with Autoflex III Smartbeam (Bruker Daltonics Inc). Differential scanning calorimetry (DSC) was carried out with a Perkin Elmer differential scanning calorimeter (DSC7) with heating and cooling rates of 10 K/min. Phase transitions were also examined by a polarization optical microscope (POM) Olympus BX51 with a T95-PE temperature-controlled THMS-600 hot stage. X-ray diffraction measurements were performed on a D8 Advance (Bruker AXS Inc.) with Cu Kα1: 1.54051 Å. FT-IR spectra were recorded with a Varian 640-IR in the range of 400–4000 cm^−1^. SEM measurement was performed with XL30 field emission scanning electron microscope.

**G1**: 2.67 g *N*-carbobenzyloxy-L-aspartic acid (10.0 mmol) was dissolved in 60 mL of DCM. 4.79 g EDCI (25.0 mmol) was added and the mixture stirred at 0 °C for 30 min. 3.72 g 1-Dodecanol (20.0 mmol) and 0.25 g DMAP (2.0 mmol) were added and the mixture stirred at room temperature for 24 h. Solvent was removed by rotary evaporation. The residue was purified by column chromatography on silica gel with methylene chloride as the eluent to afford **G1** as a white solid (5.32 g, 88%). ^1^H NMR (300 MHz, CDCl_3_, *δ*) 7.36–7.30 (m, 5 H), 5.76 (d, *J* = 8.5 Hz, 1H), 5.14 (s, 2H), 4.63 (dd, *J* = 8.7 Hz, 1H), 4.13 (d, *J* = 3.6 Hz, 2H), 4.06 (t, *J* = 6.8 Hz, 2H), 3.04–2.82 (m, 2H), 1.63–1.57 (m, 4H), 1.30–1.26 (m, 36H), 0.88 (t, *J* = 7.1 Hz, 6H). Anal. Calcd for C_36_H_61_NO_6_: C, 71.60; H, 10.18; N, 2.32. found: C, 71.83; H, 10.02; N, 2.01. *m/z* [MALDI–TOF]: 604.7 (M + H^+^).

**G2**: 1.335 g *N*-carbobenzyloxy-L-aspartic acid (5 mmol) was dissolved in 60 mL of DCM, 2.880 g EDCI (15 mmol) was added, and the mixture stirred at 0 °C for 30 min. 7.035 g L-Aspartic acid dodecyl ester (15 mmol) and 2.280 g HOBt (15 mmol) were added and the mixture stirred at room temperature for 24 h. Solvent was removed by rotary evaporation. The crude product was purified by column chromatography on silica gel with methylene chloride:ethanol (100:1) as an eluent to afford **G2** as a white solid (4.38 g, 75%). ^1^H NMR (300 MHz, CDCl_3_, *δ*) 0.89 (t, 12H, CH_3_), 1.26 (s, 72H, CH_2_), 1.62 (s, 8H, CH_2_), 2.64 (t, 2H, CH_2_), 2.81 (m, 2H, CH_2_), 2.95–3.01 (m, 2H, CH_2_), 2.93–3.01 (m, 8H, CH_2_), 4.59 (s, 1H, CH), 4.80 (s, 2H, CH), 5.13 (d, 2H, CH_2_), 6.71 (s, 1H, NH), 6.73 (s, 1H, NH), 7.29–7.36 (m, 5H, C_6_H_5_), 7.59 (s, 1H, NH). Anal. Calcd for C_68_H_119_N_3_O_12_: C, 69.76; H, 10.25; N, 3.59. found: C, 70.04; H, 10.06; N, 3.70. *m/z* [MALDI–TOF]: 1192.8 (M + Na^+^)

**G3**: 0.133g *N*-carbobenzyloxy-L-aspartic acid (0.5 mmol) was dissolved in 30 mL of DCM, 0.288 g EDCI (1.5 mmol) was added, and the mixture stirred at 0 °C for 30 min. 1.553 g of the second generation L-aspartic acid dodecyl ester (1.5 mmol) and 0.228 g HOBt (1.5 mmol) were added and the mixture stirred at room temperature for 24 h. Solvent was removed by rotary evaporation. The crude product was purified by column chromatography on silica gel with methylene chloride:ethanol (100:3) as an eluent to afford **G3** as a white solid (0.69 g, 60%). ^1^H NMR (300 MHz, CDCl_3_, *δ*) 0.88 (t, 24H, CH_3_), 1.29 (s, 144H, CH_2_), 1.59 (s, 16H, CH_2_), 2.70 (s, 8H, CH_2_), 2.83 (m, 4H, CH_2_), 2.93 (m, 2H, CH_2_), 4.02–4.12 (m, 16H, CH_2_), 4.54 (s, 1H, CH) , 4.82 (m, 6H, CH), 5.11 (s, 2H, CH_2_), 6.32 (d, 1H, NH), 7.11 (m, 2H, NH), 7.26–7.36 (m, 5H, C_6_H_5_), 7.69–7.97 (m, 4H, NH). Anal. Calcd for C_132_H_235_N_7_O_24_: C, 68.80; H, 10.25; N, 4.25. found: C, 68.55; H, 10.16; N, 4.51. *m/z* [MALDI–TOF]: 2326.8 (M + Na^+^)
